# Experimental Evaluation of Tactile Sensors for Compliant Robotic Hands

**DOI:** 10.3389/frobt.2021.704416

**Published:** 2021-10-11

**Authors:** Werner A. Friedl, Máximo A. Roa

**Affiliations:** German Aerospace Center—DLR, Institute of Robotics and Mechatronics, Wessling, Germany

**Keywords:** tactile sensors, slippage detection, hand design, grasp stiffness, grasp benchmarking

## Abstract

The sense of touch is a key aspect in the human capability to robustly grasp and manipulate a wide variety of objects. Despite many years of development, there is still no preferred solution for tactile sensing in robotic hands: multiple technologies are available, each one with different benefits depending on the application. This study compares the performance of different tactile sensors mounted on the variable stiffness gripper CLASH 2F, including three commercial sensors: a single taxel sensor from the companies Tacterion and Kinfinity, the Robotic Finger Sensor v2 from Sparkfun, plus a self-built resistive 3 × 3 sensor array, and two self-built magnetic 3-DoF touch sensors, one with four taxels and one with one taxel. We verify the minimal force detectable by the sensors, test if slip detection is possible with the available taxels on each sensor, and use the sensors for edge detection to obtain the orientation of the grasped object. To evaluate the benefits obtained with each technology and to assess which sensor fits better the control loop in a variable stiffness hand, we use the CLASH gripper to grasp fruits and vegetables following a published benchmark for pick and place operations. To facilitate the repetition of tests, the CLASH hand is endowed with tactile buttons that ease human–robot interactions, including execution of a predefined program, resetting errors, or commanding the full robot to move in gravity compensation mode.

## 1 Introduction

In real-world scenarios, robotic grasping is still a challenge due to the variation of object properties such as shape, weight, and friction, on top of the problems arising from the robotic components, for example, a vision system that cannot accurately identify a partially occluded object in cluttered scenes or a hand that cannot robustly hold the grasp when an unexpected collision happens. To robustify grasping applications, not only a well-engineered gripper or robotic hand is required. Additional sensorial information can also help enable more intelligent controllers, which can, for instance, detect slippage and adjust the gripping force accordingly. A review of available technologies for tactile sensing, including their possible advantages and disadvantages for different applications, can be found in Kappassov et al. (2015). A more recent overview on existing tactile sensors and the information that can be abstracted from them is provided in Li et al. (2020). For the specific problem of slip detection in dexterous manipulation, an overview is given in Chen et al. (2018).

Different tactile sensors provide varying performance, depending on the situation where they are used. To provide a standard baseline for comparison, in this study we evaluate a number of tactile sensors integrated on the same gripper, the CLASH 2F, based on the technology of the CLASH 3F hand (Friedl et al., 2018). For technical reasons, we restrict our analysis to the sensors that can be integrated in this gripper. We are aware that this can leave out several modalities, such as sensors specifically developed for soft hands (Wall and Brock, 2019), but we believe our selection offers a good generic overview of tactile sensing technologies available nowadays. To facilitate the interaction with the gripper and to ease teaching of poses required for the tests, we developed a user interface that can be mounted on the CLASH 2F (also on the CLASH 3F). This user interface helps to teach grasp poses of the hand and also to easily access basic control functions of the robot (e.g., gravity compensation). Furthermore, it delivers status information of the robot and the hand by providing visual feedback to the user, which greatly facilitates solving unexpected errors during operation.

For our experimental evaluation, we focused on the contact information required to provide data on normal force, rough position of the contact area, and object slippage. Normal forces can be measured by different modalities, for example with simple and unexpensive SMD pressure sensors (RightHand Robotics, 2021; Kõiva et al., 2020) or with resistive pressure sensors as in the CMOS tri-axis technique with an integrated proximity sensor (Lee et al., 2019) or even more flexible as in Büscher et al. (2015). Also, a capacitive sensor can be used to detect contacts, for instance using 3D printed sensors as in Ntagios et al. (2019). For detecting slippage, we can observe the shear and normal force and then adjust the normal force at a contact point (Ajoudani et al., 2016; Contactile.com, 2021; Tomo et al., 2018) or detect the slippage by processing and interpreting the sensor signals (Nakagawa-Silva et al., 2019; Rosset et al., 2019). Multi-modal sensors as in Ruppel et al. (2018) and Weiner et al. (2019) can provide additional information for object identification, including, for instance, shear force or temperature of the object.

To verify the applicability of the sensors to a real use case, we used a benchmark proposed in Mnyusiwalla et al. (2020) for logistic use cases. We used two benchmark scenarios involving cucumbers and punnets, where all grippers shown in Mnyusiwalla et al. (2020) failed, and we tested here if additional sensor information helps to solve the intended tasks. After trying the two selected scenarios using the CLASH 2F, we verified that now our system performs much better. For the scenario where one full layer of cucumbers fills the crate, we were able to retrieve all of the aligned cucumbers by implementing an adapted vision and planning strategy. The additional information from the implemented sensors also allows us to successfully grasp all objects in the second tested scenario, a crate filled with one full layer of punnets. The addition of tactile sensors allows us to prevent multiple failed grasps, as we use them to trigger error-coping strategies such as repositioning the gripper. We can now also detect slippage and objects that rotate inside the hand, and adapt the grasp or the arm motion accordingly.

## 2 Hardware: CLASH 2F Gripper and Tactile Sensors

### 2.1 Tactile Sensors

**FIGURE 1:**
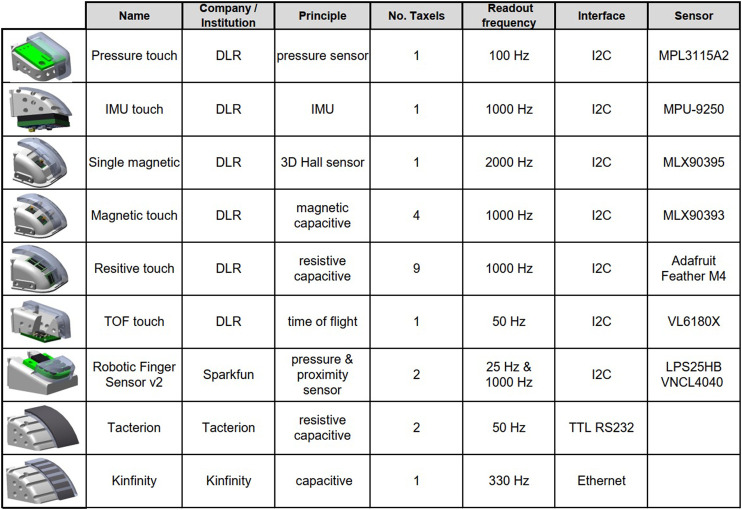
Tactile sensors used in the comparative study, including self-made and commercially available sensors.

This study compares the performance of different tactile sensors by testing several aspects, including sensibility depending on the finger stiffness and the object material, and their applicability for detection of edges and slippage. The test procedures are applied to sensors with different underlying technology, including pressure, magnetic, capacitive, proximity, or IMU-based sensors. The summary of all sensors used in this study is provided in Figure 1. The main difference to consider when using these sensors is the number of taxels they provide. Single taxel sensors tend to be more affordable, including the commercial sensors from Kinfinity, Tacterion, and Sparkfun. The Robot Finger Sensor v2 from Sparkfun (2021) was originally designed by Patel et al. (2018), based on an I2C proximity sensor molded in silicone. Now it also includes an extra pressure sensor, which helps to deal with the problem of reflections on different materials. If more human-like geometry at the fingertips is required, then sensors such as the capacitive/resistive sensor from Tacterion (2021) can be used, as it can be bent in two directions. The capacitive textile sensor from Kinfinity (2021) uses two electrodes (compared to one electrode in the sensor from Tacterion), which should reduce the electromagnetic noise from the environment. On the other hand, there are high dimensional sensors, which have changeable silicone pads, such as the resistive sensor array or the tri-axis magnetic MLX90393 sensor. In general, for a real application the fingertips should be easily exchangeable as the silicone pads must be changed with certain frequency to deal with wear and contamination.

### 2.2 Construction of the Sensors

**FIGURE 2:**
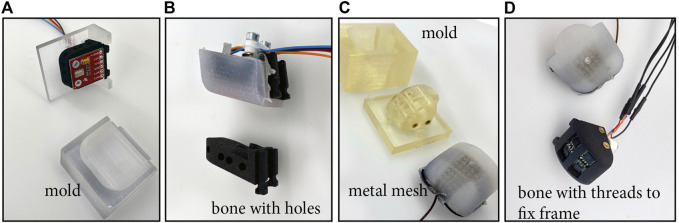
Self-made tactile sensors: **(A)** casting mold for the pressure sensor (before casting); **(B)** casted pressure sensor and “bone” (fingertip structure) with holes for simple sensors such as pressure sensors, IMU, or VL6180X; **(C)** casting mold and resulting silicone pad for the magnetic four-axis sensor; and **(D)** fingertip structure with four integrated MLX90393 sensor chips and silicone pad ready for assembly.

Besides the commercially available sensors, which are adapted to work on the CLASH 2F gripper, we decided to try out different sensor modalities using the same base hardware. We self-developed tactile sensors based on ToF (Time of Flight), IMU, and pressure, embedding the sensor and the required structure for providing a single taxel in a suitable exchangeable fingertip to be mounted on the gripper. We have empirically found out that silicone pads are more robust and give better friction properties than PU (Polyurethane) pads, as used for example in the DLR Hand II (Butterfass et al., 2001; Grebenstein et al., 2019), so we decided to use silicone for the finger pads. The transition from the soft pad material to the hard finger structure is always problematic, as the soft pad tends to detach easily from the supporting structure. A bonding agent such as *Wacker Haftvermittler HF86* can be used, and it leads to good results, as obtained, for instance, with the AWIWI II hand (Friedl et al., 2015), but the bonding agents pose a risk for human health, so we decided to try a different approach. For the single axis sensors, for example the tactile sensor based on pressure, the contact surface with the finger structures can be enhanced (in order to increase adhesion with the soft pad) using holes in the supporting part, as shown in Figure 2.

For changeable finger pads, it is not easy to increase the contact surface with the underlying structure. To solve this problem, we decided to use an underlying mesh wire that is glued to the frame and provides support to the molded polymer (Figure 2). Furthermore, the mesh wire can be soldered together and can also be connected to a wire in order to use it as a capacitive sensor. We connected this sensor to the Tacterion electronics to read out the values by the sensor bridge provided in this board. We tried to use the Tacterion electronics also for the Kinfinity sensor, but the capacity was too low to get useful values, so we used the Ethernet-based electronics from Kinfinity, with a readout frequency of 300 Hz.

### 2.3 Gripper for Experiments

To provide a unified hardware baseline for the testing and to simplify the effort for mechatronic integration of multiple sensors, we decided to use the CLASH 2*F* gripper, based on the same technology as the CLASH 3*F* hand. The fingers in CLASH 2*F* reuse the thumb modules of CLASH 3*F*, and it also has two extra DoF (Degrees of Freedom) at the base, to tilt the modules and increase the opening distance to over 260 mm in order to grasp large objects. The distal phalanx of the fingers was redesigned to allow a quick exchange of the fingertips. Furthermore, the new distal phalanx allows the integration of a sensor bridge with the Adafruit Feather M4, which collects all the data from the different sensors and transmits them to the hand controller. The hand control PCB, based on an Arduino Micro, has limited memory and CPU power, and it is not able to integrate all sensor libraries or machine learning algorithms required, for instance, for slippage detection.

**FIGURE 3:**
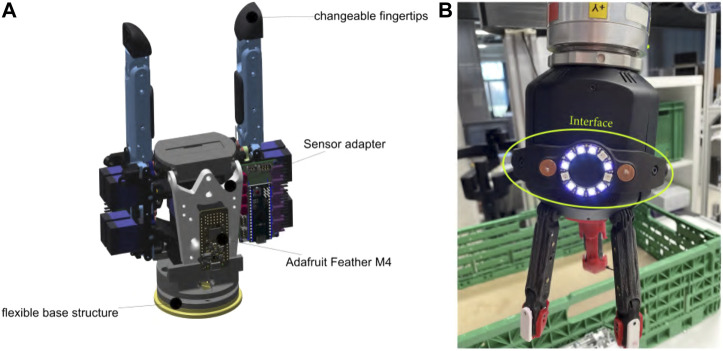
CLASH 2F gripper. **(A)** Two-fingered gripper with two thumb modules and a flexible base structure. **(B)** User interface for commanding the hand.

The gripper also includes a flexible base located right after the standard connector to the robotic arm, whose movement is measured with two Melexis MLX90935, each one of them containing 3-DoF Hall sensors (Figure 3). We included this flexible hand base inspired by previous benchmark results obtained in Negrello et al. (2020), where hands with such base, namely the hands presented in Deimel and Brock (2015); Catalano et al. (2014), had much better grasp stability in front of unexpected collisions with the environment, compared to the CLASH 3F hand. With the sensors used to measure the position of the flexible base and the information on the finger position, a single contact point on the finger can be calculated [the process is comparable to finding the contact point where a perturbation force is applied to a robot arm by using a 6-DoF force–torque sensor at the base of the robot arm (Shujun Lu et al., 2005; Iskandar et al., 2021)].

### 2.4 Gripper User Interface

A repeated use of the gripper in benchmark scenarios, such as the exemplary ones used in this study, requires a simple interface to command the hand and the robot, which should facilitate human–robot interactions, including execution of a predefined program, resetting errors, or commanding the full robot to move in gravity compensation mode. Some commercial robots or grippers include a similar interface, for example, the robotic arm from Franka Emika (2021), or the gripper from Schunk (2021). Our setup uses the KUKA LWR robot, which has no user interface, so we created a mountable one to control both arm and hand and to provide feedback to the user using LED lights, as shown in Figure 3.

The interface has four capacitive buttons, two RGB LEDs on the right and the left side, and a LED ring with twelve LEDs from Adafruit plus an Adafruit ItsyBitsy to control the LEDs and to read the button inputs. The micro-controller is connected over the I2C sensor port of CLASH, which also delivers 5V and motor power. The sensor port can be used to mount different sensors, cameras, and a wrist or a user interface on the hand.

**FIGURE 4:**
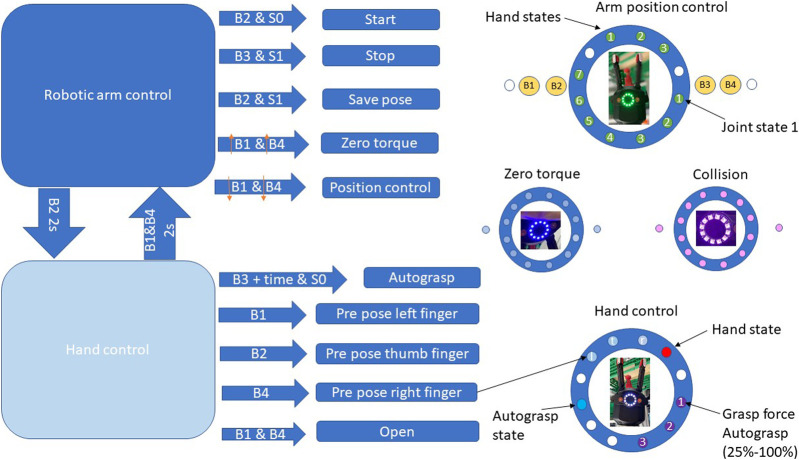
Modes of operation of the user interface in the CLASH hand. The left side shows the two operation modes and the possible button combinations to control the arm or hand. On the right side, the possible LED status information is shown. For example, for teaching one waypoint the user should press button B2 to switch on the robot, and the control state changes from S0 to S1. Now the user can press B1 and B4 for the zero-torque (gravity compensation) mode and can move the robot to a desired position. The zero-torque mode is confirmed by a blue LED ring. To save a new position, the user presses B2 and gets a short feedback with pink LEDs, to confirm that the position is saved. To grasp an object, the user has to press B2 for at least 2 s to switch to the hand control (grasp) mode. With the buttons B1, B3, and B4, the user can change the pre-grasp pose of each finger. To grasp the object the user has to press B3. The press duration changes the grasp force, as indicated by the LED ring.

To verify the interface and its behavior in practice, we used a simple test scenario. The user has to program how to grasp a punnet in the benchmark scenario P2 in Mnyusiwalla et al. (2020). A direct grasp with CLASH 3F is not possible, as a two-finger diagonal grasp is not reachable with the kinematics of the hand. Hence, the user has to teach the robot a motion that separates the desired object from the other ones, so that enough space is available around the object to be able to grasp it. We compared the programming of this sequence with the input device against a GUI-based programming with a mouse and a screen at the robot setup. For the task of kinesthetic teaching to grasp one punnet with the user interface, we get three trials with an average time of roughly 35 s. However, the same task using the GUI for switching between modes of the robot and the hand takes around 60 s. These pilot tests showed that an optical feedback to indicate the user if the actual frame is saved (by activating during 0.1 s the pink LEDs in the ring) greatly improves the handling and programming of the system. Figure 4 shows the different modes of operation of this user interface.

## 3 Sensor Tests

This section presents the testing procedures performed to identify how the finger stiffness and the object material might affect the sensor sensibility, and then how the sensors can be used to detect slippage and edges, which are two critical features to enable more robust robotic grasping procedures.

### 3.1 Analysis of Sensor Sensibility Depending on the Finger Stiffness

**FIGURE 5:**
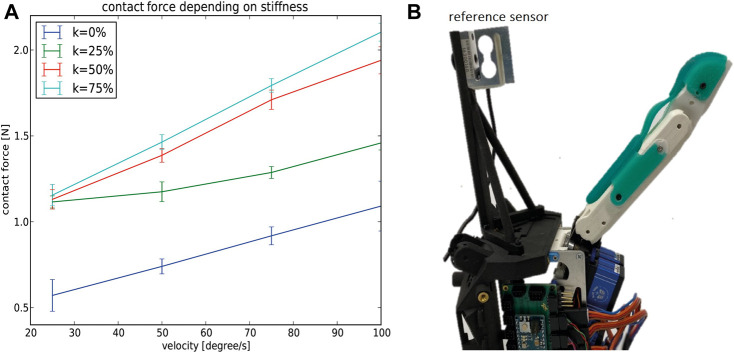
Sensor sensibility depending on finger stiffness. **(A)** Contact force depending on stiffness and finger velocity for the CLASH 3F thumb. **(B)** Testbed with the thumb, and a fixed resistive sensor array.

To get an initial insight into the behavior of the sensor sensibility depending on the joint stiffness, we used the thumb module of the CLASH 3F hand and a fixed 3 × 3 resistive sensor array to measure forces applied by the thumb (Figure 5). We tested four different pretension values *k*, which directly correlate with the finger stiffness: 100% pretension is equal to 100% of the maximum actuator force. With the known coupling matrix for the finger (Friedl et al., 2018), its null space, and the force-to-stiffness relation for the FAS (Flexible Antagonistic Springs, Friedl and Roa, 2020), the corresponding joint stiffness can be obtained. For the test, we moved the thumb toward the sensor at different velocities, stopping the test when we detected the contact with the touch sensor. Figure 5 shows the results of this initial test. Note that the contact force increases with higher stiffness. With lower stiffness, the finger can compliantly adapt to the contact and the applied force builds up at a slower rate, thus the force is lower when the contact is detected. For our intended use cases, we require high sensibility to low contact forces, so we focus our subsequent test only on low pretension values.

**FIGURE 6:**
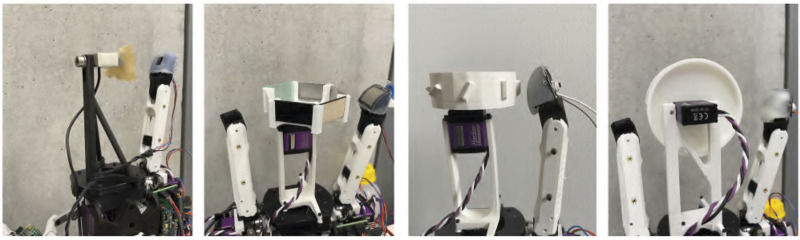
Testbeds for verification of the tactile sensors on the CLASH 2F. **From left to right**, testbeds for: sensibility depending on stiffness, sensibility depending on surface materials, edge detection, and slippage detection.

The testbed for the next test follows the same principle, but now using the CLASH 2F, as shown in Figure 6. The contact force was measured by a KD24s sensor from ME-Messsysteme, which includes a 2 N force gauge. We used two pretensions and different joint velocities and repeated the test 10 times for each combination. We then calculated the medium and standard deviation of the detected contact force. The pretension of zero is equal to a joint stiffness of 0.23 Nm/rad in the base joint and 0.12 Nm/rad in the distal joint. If we increase the pretension to 12.5*%* we get stiffness of 0.34 Nm/rad and 0.16 Nm/rad in the base and distal joint, respectively. The maximum joint stiffness is 1.04 Nm/rad without any load on the finger.

The procedure to perform this test is as follows:

1) Place the finger at the start position, 2 cm away from the sensor.
2) Move the finger with an angular velocity of 5°/s toward the force sensor and stop when the force sensor reaches a detected force of 10 mN, save the finger position as *cp*.
3) Now repeat the open-close cycle with a threshold of 50 mN and record the sensor signal of the tested tactile sensor.
4) Analyze the recording to get the minimum threshold for each sensor. The threshold should be higher than two times the noise
5) Now increase the velocity to 25°/s and drive the finger toward contact until it reaches the tactile sensor threshold.
6) Check if the finger position is equal or larger than *cp*; if true then save the sensor threshold, and if not then save as result 5 N (a high value artificially selected) to punish sensor failures.
7) Repeat the previous step 10 times and then increase the angular velocity with increments of 25°/s, repeat this sequence until reaching 175°/s.
8) Increase the stiffness of the finger, if possible, and repeat the previous steps.

**FIGURE 7:**
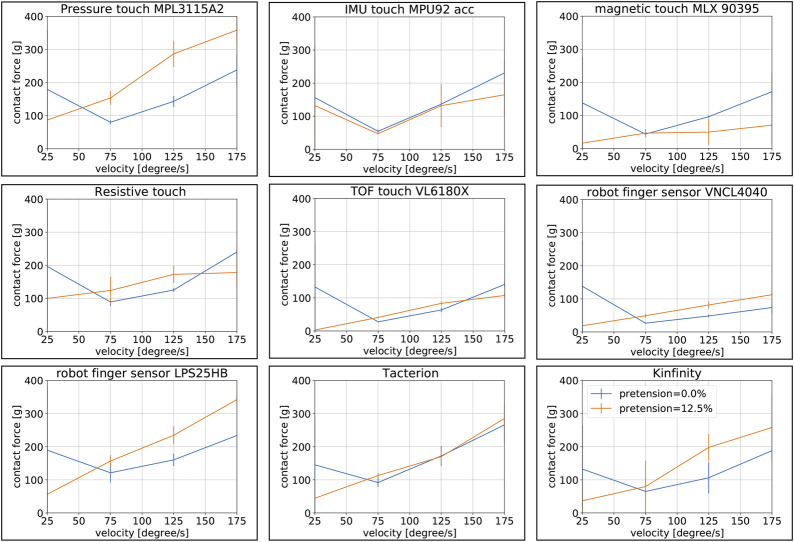
Detected contact force for all nine sensors depending on joint stiffness and joint velocity. The blue line shows 0% pretension (corresponding to a stiffness of 0.23 Nm/rad and 0.12 Nm/rad in the base and distal joint, respectively) and the orange line 12.5% pretension (corresponding to a stiffness of 0.34 Nm/rad and 0.16 Nm/rad in the base and distal joint, respectively).

The visual summary of results is provided in Figure 7. Note that for increasing angular velocities, the applied contact force increases, but on some sensors a higher stiffness can reduce the detected contact force for high joint velocities such as for VL6180X, or with a stronger effect on MLX90395. Note that for higher stiffness the finger stops faster, because the finger is not overshooting so much (going beyond the stop position *cp*) and it is also storing less energy in the springs. Sensors with high readout frequency such as the resistive sensor MLX90395, or the proximity sensor VL6180X can cope better with this problem, as they will quickly detect the increase in contact force and trigger earlier the stop of the finger. Note also that the IMU does not work so well for low velocities, which can be seen on the high values obtained at these low velocities. Due to the low impulse at the contact, the value is below the threshold of the sensor and the FAS has to stop the finger. The lowest contact force of around 2 g can be achieved by VL6180X, followed by VNCL4040, and then comes the capacitive mesh with around 3 g of detected contact force. The Kinfinity sensor also behaves quite well, but for low stiffness and low velocity the sensor generates too many false-positives, which come from unfiltered peaks in the measurement. If we use the torque sensor values based on the measurement of the FAS to stop the finger, we can reach around 30 g, which agrees with the result from Friedl and Roa (2020).

### 3.2 Analysis of Sensor Sensibility Depending on the Surface Material

Another interesting aspect to evaluate is the behavior of the sensors when grasping objects made out of different materials, which happens very often in real applications. Naturally, it is expected that the optical sensor will have problems with transparent or highly reflective materials. Also, the magnetic sensor can react properly mainly for magnetic materials such as steel. To test this aspect, we created a testbed that uses a wheel where small plates of different materials can be inserted for testing (Figure 6). In our tests, we used six materials: magnetic steel, non-magnetic steel, white PLA (polylactide), black PLA, wood, and a transparent acrylic glass (PMMA - polymethyl methacrylate). As a reference sensor to generate the ground-truth contact force, we used the FAS of the hand, which makes the testbed simpler, but the results are less accurate than those obtained with the force sensor used in the previous case. The finger is commanded to close and touch the plate. The forward kinematics of the finger indicates if the finger should be already in contact with the plate; if the contact is missed, we artificially punish this with a high contact force value. The procedure for this test includes the following steps:

1) Place the finger at the start position, 2 cm away from the insert.
2) Move the finger with an angular velocity of 5°/s toward the plate and stop when the FAS sensor reaches a force of 30 mN, save the finger position as *cp*.
3) Now increase the velocity to 50°/s.
4) Drive the finger in contact until it reaches the tactile sensor threshold (determined in the previous test).
5) Check if the finger position has reached or surpassed *cp*; if true then save the sensor threshold, and if not then save 5 N as result to punish sensor failures.
6) Repeat the previous two steps 10 times.
7) Rotate the wheel for using the next material plate, and repeat the procedure until all materials are tested.

**FIGURE 8:**
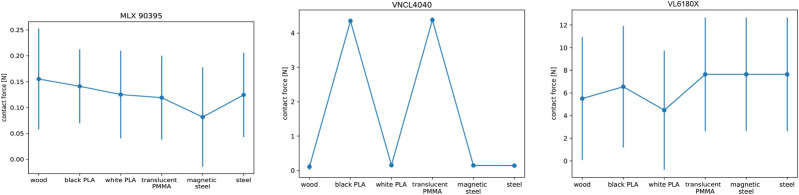
Contact force depending on the material, for the magnetic, proximity and ToF sensors (left to right).

For the VL6180X, this procedure unfortunately does not work properly, as the contact force can be very low, and it will not be detectable with the FAS. Figure 8 shows the results for the two optical sensors and the magnetic one; the rest of the sensors do not react differently for different materials. All tests were performed with a joint velocity of 50°/s and a stiffness given by 12.5% pretension. VL6180X has problems with highly reflective materials such as steel, or with translucid materials as the transparent acrylic glass, which results in a sensor error not handled by the control, and the wrong sensor value indicates the controller that there is a contact when there is actually none. For future use of the sensor, the error bit has to be readout and the control has to switch to a different sensor modality to sense the contact. VNCL4040 also has problems with the transparent material and with the black PLA, which generates a signal too low to stop the finger. The control stateflow stops the finger if the FAS torque reaches more than 0.4 *Nm*, as observed in the results of Figure 8.

### 3.3 Analysis of Slippage Detection

For handling a large variety of products differing in weight, size, and stiffness, adapting the grasping force to provide just the required force to grasp the object while avoiding slippage is paramount. To verify the slippage detection with the different sensors, we created a testbed with a plastic wheel made of white PLA, shown in Figure 6, which contains surfaces with different roughness to test if a given sensor can detect slippage or not. The slippage detection requires, in general, a readout frequency higher than 50 Hz, as demonstrated in Holweg et al. (1996). Hence, we did not test the Tacterion sensor nor all pressure sensors, as they cannot be read out at that high frequency. The magnetic three-axis sensor can detect slippage before it happens, by observing the proportion between normal force and shear force. For the other sensors we can analyze the signal by Fast Fourier Transform (FFT) or with Discrete Wavelet Transform (DWT) to detect slippage. All sensors were tested 30 times with different slip velocities and positions on the wheel. The internal torque sensing of the finger was used to set the contact force against the wheel. The steps followed in the test are given below:

1) Place the finger at the start position, 2 cm away from the wheel.
2) Activate the friction wheel to rotate randomly between 0 and 360°.
3) Drive the finger in contact, with a contact force of 2 N.
4) Start to log the tactile sensor values.
5) The friction wheel starts to rotate with a random velocity from 5 to 75°/s.
6) Save the logged data.
7) Repeat all the previous steps until completing 30 trials.

**FIGURE 9:**
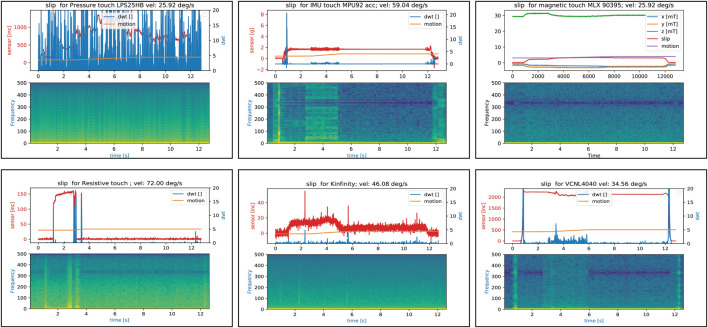
Slippage detection using different sensors. For each sensor we show the raw sensor signal, Discrete Wavelet Transform (DWT) and the motion of the wheel in one plot, and a spectrogram plot of the sensor signal in the plot below. Note that for MLX90395 instead of the DWT we show a slip signal, based on Eq. 1. A high response in the spectrogram indicates that the signal can be well detected using the DWT, for example, for the IMU, which shows a clear response when the wheel starts to rotate. On the other hand, the Kinfinity signal is too noisy to differentiate slip from noise.

Figure 9 shows an example of the results. As expected, the IMU and MLX90395 work well for different velocities; also VNCL4040 can detect slip quite well using DWT. The IMU sensor works on all surfaces of the wheel, while for MLX90395 the contact point should be near the sensor to get a suitable signal. This also applies to the VNCL4040 sensor, where the detection is influenced by the optical properties of the silicone and the object. For a more demanding application, we would have to consider a sensor enclosed in an opaque medium at the outer hull of the pad in order to obtain a better force signal, independently of the object material. The Kinfintiy and the pressure sensor fail in this detection due to the high noise in the signal (filtering the signal did not help). The resistive sensor works in some occasions, but not in all 30 trials. This can be explained by the construction of the sensor: the sensing electrodes lie on a flat PCB, and the resistive material is a plastic sheet of *velostat*, which is glued to the PCB. The silicone pad above the PCB has a 3 × 3 structure, which mimics the form of the electrodes to reduce crosstalk. The perceived signal strongly depends on a uniform force transmission by the silicone, which is not guaranteed by this construction. A better sensor for this purpose would be filled up with a thin silicone film first, to mount afterward the exchangeable pad above.

Using the information of the detected slip, we can integrate this detection in the automatic grasp state machine of CLASH using the following steps:

1) Bring all fingertips in contact with the object, verifying the contact with the finger torque sensors and fingertip tactile sensors.
2) Lift the object, while analyzing normal and shear force.
3) If shear forces get higher than the limit permitted by the normal force, increase (stepwise) the normal force until no more slippage is detected (or until the force cannot be further increased). The required torque at the motors is computed using the Jacobian matrix for the contact position.
4) When an “open” command is given, stop slippage observation and open the hand.

The detection of slippage uses, as an underlying assumption, a simple friction cone model (Wettels et al., 2009); slippage occurs if the tangential force (vectorial sum of *t*_*x*_ and *t*_*y*_) exceeds the normal force (*t*_z_) times the friction coefficient (μ_f_). The factors *c*_*x*_, *c*_*y*_ and *c*_*z*_ are calibration factors in this expression for the non-slippage condition:

$$1<\mu_{F}\frac{c_{z}t_{z}}{\sqrt{c_{x}t_{x}^{2}+c_{y}t_{y}^{2}}}$$ (1)

which assumes that the normal force (z-component) of the sensor is the dominant one. However, if the contact is far away from the center of the sensor, as it could happen for instance with MLX90395 embedded in a round fingertip, another component of the force can actually dominate the sensor output and the slippage model will lead to inaccurate results. A workaround would be adjusting the coordinate frame so that the *z*-axis is aligned with the highest force direction after the initial grasp, or building a flatter finger pad to guarantee that the z component is always dominant in the sensor output using the initial coordinate frame. Also, re-orientating the finger to achieve a contact point directly over the sensor would help. For our final benchmark in the next section, we decided to use a fingertip with a flatter pad.

### 3.4 Analysis of Edge Detection

Another aspect highly relevant for a tactile sensor is its ability to differentiate geometric features. For a grasping application in logistics, for instance, the detection of edges can help to secure difficult objects. Another case happens if an object is rotating within the hand after the grasp is performed; the grasp is not anymore the nominal one used for collision-free path planning, and the object might hit obstacles during transportation. The early detection of the rotation helps to adjust the path or to readjust the grasp using external forces (Chavan-Dafle et al., 2014). Note, for instance, that for a cylindrical object, the sensor perceives a similar signal when grasping the cylinder along its length or when touching an edge. We implemented a testbed, shown in Figure 6, where a wheel with edges on its periphery is mounted on the central part of the hand. The wheel has 8 edges, oriented in 22.5° steps and with a height of 5 mm above the wheel. The test procedure for the edge detection has the following steps:

1) Place the finger at the start position, 2 cm away from the sensor.
2) Choose a random edge on the edge wheel.
3) Drive the finger toward contact with a contact force of 3 N.
4) Log the tactile sensor values.
5) Repeat 20 trials per edge.

**FIGURE 10:**
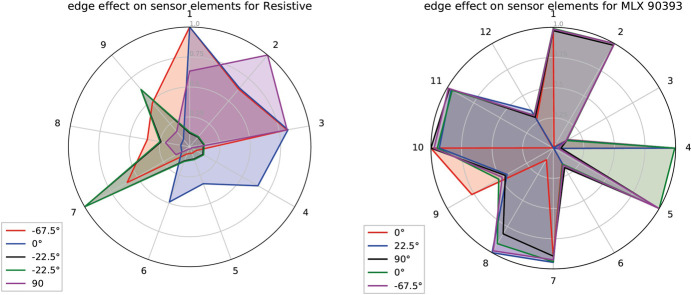
Radar plot to show the edge effect on the different sensor taxels for the resistive and MLX90393 sensors.

We tested both multi-taxel sensors, the 3 × 3 resistive, and the sensor with the four MLX90393, which would be capable of differentiating the edges thanks to their higher spatial resolution (resistive sensor: 2 *mm*, MLX: 10 mm). The results obtained with the procedure described above can be nicely visualized in a radar plot, as shown in Figure 10. Unique shapes of the areas guarantee that the edges can be discerned quite well; also, the plots help to recognize the sensor that best identifies the edges in different orientations. Note that with the measurements of the four MLX90393, it is hard to discern the different edge orientations, as the resulting areas are quite similar for most of the tested orientations. The test was run with a threshold value of 0.3 Nm for the base torque sensors of the fingers, and repeated with all edges on the wheel. The resistive sensor array shows clear signals over multiple trials for the different edges. Machine learning approaches could help for generalizing the detection for random orientations of the edge.

### 3.5 Multi-Modal Sensors

**FIGURE 11:**
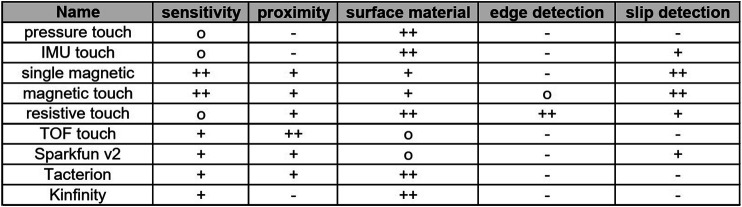
Summary of performance characteristics for all the tested sensors.

From the previous tests, we can observe that different sensors have different strengths, and some of the sensors cannot even be applied to some of the tests. An overview of all sensors and their main performance characteristics is shown in Figure 11. This table can be used to guide the construction of a multi-modal sensor for different scenarios For instance, optical sensors have problems with different materials and should be not used alone, but they deliver interesting information for pre-contact detection. Also, the capacitive sensors can be used as proximity sensor, as they are also quite sensitive for this application. The mesh capacitive sensor, for instance, is simple to build and relatively easy to read out, and the mesh stabilizes the silicone pad. VL6180X provides a more precise distance measurement, which can be helpful to use it as a scanning sensor for pre-grasp adaptation. Due to the results of the edge detection test, it is clear that a suitable spatial resolution is required to properly detect object rotations; the tested commercial sensors would have to be adapted for this application by asking the vendors for a sensor with better resolution. We decided to focus on the resistive sensor for detecting the contact position and orientation, in combination with one or more MLX90395 as shear force sensor. Complemented with the IMU as fingertip orientation and slip detector, we could then create a multi-modal fingertip that provides all the required information to robustify a grasp execution. VNCL4040 could be still an option for slip detection and proximity; however, it requires larger space for integration, especially if we want to generate a multi-modal fingertip adaptable to the CLASH gripper.

## 4 Benchmark Test

**FIGURE 12:**
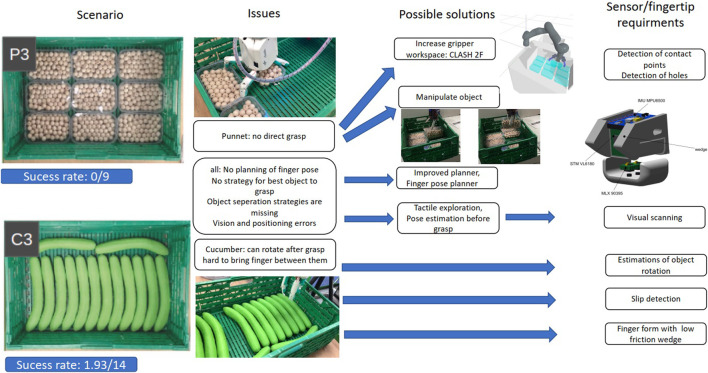
Derivation of requirements from the benchmark tests by analyzing the original failures for the study from Mnyusiwalla et al. (2020) for a crate completely filled with punnets (scenario P3) or cucumbers (scenario C3).

To test the applicability of the selected sensors, we use a published benchmarking framework for pick-and-place tasks, designed originally for the logistic domain (Mnyusiwalla et al., 2020). It entails picking up fruits and vegetables from a container and placing them in an order bin. The original benchmark proposed scenarios with different degree of difficulty. In particular, two of the scenarios, named *C*3 and *P*3, were not solved by any gripper or hand in the original study. Those scenarios, depicted in Figure 12, deal with grasping objects from a crate, where the crate is completely filled with cucumbers (scenario *C*3) or punnets (scenario *P*3).

First, we revisited the initial results of the benchmark, analyzing the root causes for failure, in particular for the CLASH hand, as shown in Figure 12. Some of the identified problems, mainly the lack of a local planner for finger poses in such a cluttered environment, were recently solved in Sundaram et al. (2020). The main challenge in the punnet scenario *P*3 is that a direct grasp of the objects is not possible. The difficulty is greatly reduced after the first punnet is retrieved, but this requires some smart sequence of steps, for example, find a hole between the punnets, place the thumb in the hole, move the hand toward the middle of the punnet to separate them, tilt the punnet using a suitable arm motion, reaccommodate the hand to a new grasp pose, and grasp the punnet. In such a complex manipulation sequence, if any motion fails, a state estimation based on the sensors would improve the success rate, as it would promptly call for a corrective action or a replanned strategy. For the new CLASH 2F, we set up a PyBullet^1^ simulation (Figure 12) to verify if it is possible to grasp the punnet on the long side, or even diagonally, to be able to grasp it directly without any complex sequence. In fact, both options are possible due to the hand kinematics, which allows the hand to adapt and grasp light objects up to a size of 250 mm (the punnet dimensions are 115 mm x 175 mm). However, the diagonal grasp is hard to stabilize, but this would be in principle possible using, for instance, the MLX90395 sensors, as we would be able to measure in which direction the punnet slips and then adapt the position of the fingers. This approach needs a signal on the MLX90395 sensors, which are in the middle of the finger pad, to detect the rotation angle of the punnet. A feasible strategy is then a combination of direct access on the long side of the punnet and then a simple move to come between the punnets and grasp one of them. For the scenario *C*3 with the cucumbers, also the first grasp is critical, and it is the hardest one to get, as there is little space for inserting a finger for the first grasp. This could be improved with a more wedged finger. We developed a first prototype of such new fingertip, as shown in Figure 12. The fingertip includes multiple sensor modalities, and follows the ideas presented in Section 3.5. VL6180X helps find the hole between the punnets and the correct fingertip position for the cucumbers. Including an IMU helps to detect slippage of an object, but can also give a state estimation of the fingertip position, which helps the planner to decide if the planned step was reached or a relocation of the finger is necessary. The two MLX90395 sensors can detect slippage and deliver more sensitive touch information.

The first prototype of this fingertip uses a commercially available sensor PCB leading to a bulkier design, which makes it difficult to reach the limited spaces between the objects in the desired scenarios, but allows us to try out the design. We used it first in the *P*3 scenario. For detecting the objects we used a single-shot multi-box detector as in Sundaram et al. (2020). The finger pose planner, which we also introduced in that study, was adapted for the two-fingered CLASH 2F for delivering poses such as the diagonal grasp required for the punnet case, as shown in Figure 14B. It can also generate the initial pose of the fingers to slide between the punnets. However, due to our bulky prototype this strategy is not implementable now, as it would destroy the punnets. Therefore, we first tried our fingertip prototype on the *C*3 scenario, with the cucumbers.

### Scenario C3: Crate Full of Cucumbers

**FIGURE 13:**
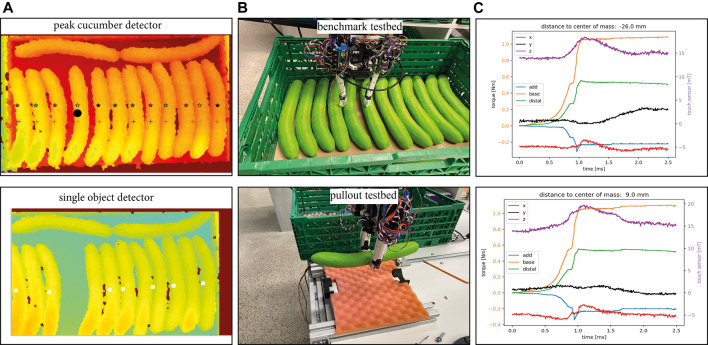
Scenario *C*3. **(A)** Vision algorithms. **(B)** benchmark testbed and pullout testbed. **(C)** Output of the MLX90395 sensor during pullout tests, where the y-component (black line) clearly signals if a cucumber rotates inside the hand. The orange, green, and blue lines (with corresponding scale in the left *y*-axis) show the finger joint torques; the object rotation cannot be detected by them, as they do not change after the rotation starts. The red, black and magenta lines (with corresponding scale in the right y-axis) show the response of the tactile sensor in the three directions x, y and z.

A new vision algorithm was implemented to detect the cucumbers, as the single-shot multi-box detector and the watershed algorithm from Sundaram et al. (2020) were not able to detect all cucumbers (Figure 13). The decision on the first object to retrieve should be based on the object that has most space around it, which for the original *C*3 scenario are the two transversely lying cucumbers. However, we decided to focus on the more challenging case of twelve aligned cucumbers. The vision algorithm scans two lines in the depth image and searches for the peaks, which represent the top of the cucumbers. This simple approach indicates the distances between the cucumbers and the orientation angle and identifies the object with most space around it, which will be the first one to be grasped. It is clear that this algorithm works with the prior knowledge of the use case, and assuming that the cucumbers are all aligned. We tried this process two times with the full box of cucumbers. In the first try we were able to get out the first cucumber, we retrieved 10 of 14 cucumbers in a single sequence. The second try failed at the first cucumber. We observed that the force used to poke between the cucumbers was too low, so we check with the IMU if the distal phalanges are straight and then if the distance to the ground is small enough based on VL6180X; if not, we increased the push force and tried it again until the finger gets between them or a push force threshold is reached. If the distal phalanx of one finger is tilted we change the position of the finger according to the signal. Furthermore, we can check if the fingertip is looking toward the valley between two cucumbers or not, and adapt the position of the fingers accordingly. This implementation allowed us to grasp all cucumbers.

**FIGURE 14:**
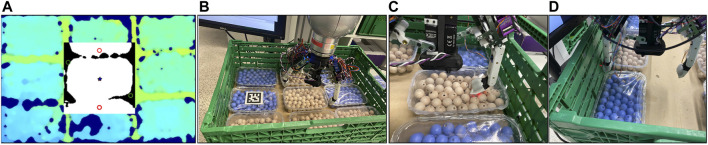
Scenario *P*3. **From left to right:** **(A)** finger position planner output, **(B)** CLASH 2F pose to grasp the first punnet, **(C)** finger failed to push the punnet away from the wall, **(D)** second push strategy with the help of the free space around the objects.

In some initial trials, we lost a cucumber in the transport phase, as the gripper did not grasp the object at the center of mass (CoM) and the cucumber rotated within the hand; the object later hit the wall of the crate and fell down. The shear force sensor helps to detect such cases, for example, if the cucumber is sliding out or if it is rotating after the lift. Adding a resistive array in front of MLX90395 is planned, which would help to get more clear information, but this needs a new PCB design. We created a pullout testbed (Figure 13) to verify if MLX90935 helps to detect the described failure. The testbed is based on a stepper motor with a winder and a FAS element to control the pull force on the grasped object. The pull tendon has a thread terminal, which allows connecting it to test objects. We inserted the thread in the CoM of one plastic cucumber and tested if we could identify the rotation in the signals obtained with the MLX90395. Figure 13 shows the results for two different grasp distances to the CoM. The y-component clearly indicates in which direction the cucumber is rotated after the arm has lifted it at around 5 cm. If the cucumber stays in horizontal direction after the lift, the y-component remains unchanged. This sensorial information allows us to adapt the path of the robot to prevent a collision between the object and the environment, and it can also provide valuable information for a suitable placement of the object.

### Scenario *P*3: Crate Full of Punnets

With the results from the cucumber experiments, we tried again the punnet scenario. A good strategy for the first punnet is to use a diagonal grasp with very low contact force by the FAS and high stiffness on the fingers, which allows us to place the fingertip against the border of the punnet to rotate it. Using the ground distance estimation with the VL6180X and the combination of IMU and distal torque to check if the last phalanges are straight, we are able to grasp the first punnet in a quite reliable manner. The same sensor information can also be used for the next punnets, but instead of grasping in diagonal we try to push them away from the walls. For this, we sense by tactile exploration the contact with the wall using the torque signal at the base joint of the finger. The IMU could also provide such information. Then we go down until we reach a contact force of 5 N to avoid destroying the punnet or its content. Afterward, we analyze the pose of the finger’s distal joint (Figure 14C). If the finger is not able to push the punnet away from the wall, we lift the hand, sense the wall again, and then go to half punnet width in direction to the middle of the crate (Figure 14D). The next step is to go down and then back to the starting pose, which pushes the punnet away from the wall easier than from the top. Due to the shape of the punnets and the diameter of the fingers, MLX90395 can barely identify a contact, but it helps in the case that the punnet is rotating inside the hand after it is grasped. We tried the benchmark multiple times and were able to grasp 9 out of 9 punnets. Naturally, the strategies implemented so far have been manually chosen, depending on the object’s pose inside the crate and on restrictions of the arm, but this should be integrated in a suitable manipulation planner.

Besides the special problems we solved for the two particular scenarios presented here, for example a special vision pipeline for the cucumbers, the selected tactile sensors will improve the results for all other objects in the benchmark in Mnyusiwalla et al. (2020), as they will allow for instance precise pre-position of the fingers in the cluttered crate if necessary. Slip detection will help to find the lowest possible grasp force and reduce damages or lost objects during transportation. The object orientation is also an interesting feature for handling all objects in the benchmark, to adapt the grasping force if the object rotates inside the hand, to perform the transportation of the object without collisions by adapting the path to the new pose of the object inside the hand, or by reacting properly in case an unexpected collision happens. Furthermore, the picks per hour will be increased as the planner can react much earlier on potential failures using the additional sensorial information, which saves unnecessary robot motions and hence execution time.

## 5 Final Discussion

This study presented the tests of different tactile fingertip sensors and their performance in combination with a variable stiffness finger. We proposed test procedures to verify aspects such as sensitivity of the sensors depending on the finger’s stiffness and the object material, and proposed a novel testbed and a procedure for testing slippage detection and edge detection. The CAD models of the testbeds and corresponding software for the test procedures are available upon request.

Our results indicate that low joint stiffness and high readout frequency improve the sensibility to grasp objects with low gripping forces. Also, proximity sensing based on capacitive or optical sensors helps to reduce the velocity before a contact actually happens. We tried out nine different sensor types, which were mounted on adapted fingertips and can be easily interchanged on the newly introduced two-fingered gripper CLASH 2F. The gripper is based on the thumb modules of CLASH 3F, and has two extra DoF, to tilt the modules and increase the opening distance in order to grasp wide objects. To facilitate the programming and interaction of the gripper, we developed a generic user interface for grippers, which allows us to control both the gripper and the robot arm and record any desired motions for later use in automatic manipulation sequences. Furthermore, the interface gives the user optical feedback about the state of the arm and the hand, to quickly solve potential problems in production.

As a test case to verify the benefits of the tactile sensors, we used the established benchmark in Mnyusiwalla et al. (2020), where multiple grippers failed in the most challenging scenarios. Thinking on this use case, we decided to create a multi-modal fingertip that integrates IMU, a time of flight sensor VL6180X, and two 3-axis magnetic sensors MLX90395. Such multi-modal combination allows an effective implementation of complex manipulation strategies that exploit environmental constraints to solve difficult grasping tasks. For instance, the sensors are used to identify wrong pre-grasps, or to detect an object that rotates inside the hand, which requires some replanning action. Furthermore, the sensors can detect slippage and therefore allow adaptation to grasping objects of different weights and rigidity, which is a mandatory characteristic to solve grasping of a large variety of objects in the logistic industry, where suction cups do not always work for all objects. The results are mainly sensor-centric, so they are extendable to other robot fingers and use cases, and can also be extrapolated to soft or continuum manipulators (e.g., the RBO III hand; Bhatt et al., 2021) with enough space on the phalanges to mechanically integrate the sensors. Note that the focus of the study was on the analysis of the sensor performance and its application on the benchmark use case. The automatic planning of such complex manipulation actions was out of the scope of this study, but it is certainly an interesting aspect to explore in the future.

## Data Availability

The raw data supporting the conclusions of this article will be made available by the authors upon request.

## Footnotes

1
http://pybullet.org
